# Clinico-pathological and epigenetic heterogeneity of diffuse gliomas with *FGFR3::TACC3* fusion

**DOI:** 10.1186/s40478-023-01506-z

**Published:** 2023-01-16

**Authors:** Alice Métais, Arnault Tauziède-Espariat, Jeremy Garcia, Romain Appay, Emmanuelle Uro-Coste, David Meyronet, Claude-Alain Maurage, Fanny Vandenbos, Valérie Rigau, Dan Christian Chiforeanu, Johan Pallud, Suhan Senova, Raphaël Saffroy, Carole Colin, Myriam Edjlali, Pascale Varlet, Dominique Figarella-Branger, The Biopathology RENOCLIP-LOC network, A. Rousseau, C. Godfraind, G. Gauchotte, K. Mokhtari, F. Bielle, F. Escande, F. Fina

**Affiliations:** 1 GHU Psychiatrie et Neurosciences, Site Sainte-Anne, service de Neuropathologie, Paris, France; 2 Institut de Psychiatrie et Neurosciences de Paris (IPNP), UMR_S1266, INSERM, Equipe IMA-BRAIN (Imaging Biomarkers for Brain Development and Disorders), Université de Paris, Paris, France; 3 APHM, CHU Timone, Service d’Anatomie Pathologique et de Neuropathologie, Marseille, France; 4 Aix-Marseille Univ, CNRS, INP, Inst Neurophysiopathol, Marseille, France; 5 Department of Pathology, Toulouse University Hospital, Toulouse, France; 6 Groupe Hospitalier Est, Département de Neuropathologie, Hospices Civils de Lyon, Bron, France; 7 Claude Bernard University Lyon 1, Lyon, France; 8 Department of Cancer cell plasticity – INSERM U1052, Cancer Research Center of Lyon, Lyon, France; 9 Department of Pathology, Lille University Hospital, Lille, France; 10 Department of Neuropathology, Hôpital Pasteur, Nice, France; 11 Department of Pathology, Gui de Chauliac Hospital, Montpellier University Medical Center, Montpellier, France; 12 Service d’Anatomie et Cytologie Pathologiques, Pontchaillou University Hospital, Rennes, France; 13 Department of Neurosurgery, GHU Paris Psychiatrie et Neurosciences, Paris, France; 14 Departments of Neurosurgery and Psychiatry, Assistance Publique-Hôpitaux de Paris (APHP) Groupe Henri-Mondor Albert-Chenevier, Créteil, France; 15 Department of Biochemistry and Oncogenetic, APHP, Paul-Brousse Hospital, Villejuif, France; 16 Department of Radiology, APHP, Hôpitaux Raymond-Poincaré and Ambroise Paré, DMU Smart Imaging, U 1179 UVSQ/Paris-Saclay, GH Université Paris-Saclay, Paris, France; 17 Laboratoire d’imagerie Biomédicale Multimodale (BioMaps), CEA, CNRS, Inserm, Service Hospitalier Frédéric Joliot, Université Paris-Saclay, Orsay, France

**Keywords:** *FGFR3:TACC3* fusion, DNA-methylation profiling, 2021 WHO classification of CNS tumours, Glioblastoma, Pediatric low grade glioma

## Abstract

**Background:**

Gliomas with *FGFR3::TACC3* fusion mainly occur in adults, display pathological features of glioblastomas (GB) and are usually classified as glioblastoma, *IDH*-wildtype. However, cases demonstrating pathological features of low-grade glioma (LGG) lead to difficulties in classification and clinical management. We report a series of 8 GB and 14 LGG with *FGFR3:TACC3* fusion in order to better characterize them.

**Methods:**

Centralized pathological examination, search for *TERT* promoter mutation and DNA-methylation profiling were performed in all cases. Search for prognostic factors was done by the Kaplan–Meir method.

**Results:**

*TERT* promoter mutation was recorded in all GB and 6/14 LGG. Among the 7 cases with a methylation score > 0.9 in the classifier (v12.5), 2 were classified as glioblastoma, 4 as ganglioglioma (GG) and 1 as dysembryoplastic neuroepithelial tumor (DNET). t-SNE analysis showed that the 22 cases clustered into three groups: one included 12 cases close to glioblastoma, *IDH*-wildtype methylation class (MC), 5 cases each clustered with GG or DNET MC but none with PLNTY MC. Unsupervised clustering analysis revealed four groups, two of them being clearly distinct: 5 cases shared age (< 40), pathological features of LGG, lack of *TERT* promoter mutation, *FGFR3*(Exon 17)::*TACC3*(Exon 10) fusion type and LGG MC. In contrast, 4 cases shared age (> 40), pathological features of glioblastoma, and were *TERT*-mutated. Relevant factors associated with a better prognosis were age < 40 and lack of *TERT* promoter mutation.

**Conclusion:**

Among gliomas with *FGFR3::TACC3* fusion, age, *TERT* promoter mutation, pathological features, DNA-methylation profiling and fusion subtype are of interest to determine patients’ risk.

**Supplementary Information:**

The online version contains supplementary material available at 10.1186/s40478-023-01506-z.

## Introduction

The current World Health Organization (WHO) classification of Central Nervous System (CNS) tumors classifies diffuse gliomas into three groups: adult-type diffuse gliomas, pediatric-type diffuse low-grade gliomas (LGG) and pediatric-type diffuse high-grade gliomas [21]. The terminology “adult-type” versus “pediatric type” means that the most frequent age of onset occurs in adult versus pediatric setting, respectively, but some exceptions might occur. Among each group, several tumor types are recorded, defined by essential and desirable criteria resulting from the combination of histopathological, genetic and/or epigenetic data [21]. Adult-type diffuse gliomas include astrocytoma, *IDH*-mutant, oligodendroglioma, *IDH*-mutant and 1p/19q-codeleted and glioblastoma, *IDH*-wildtype whereas four tumor types are recorded in the group of pediatric-type diffuse LGG: diffuse astrocytoma, *MYB* or *MYBL*-altered, angiocentric glioma, polymorphous low-grade neuroepithelial tumor of the young (PLNTY) and diffuse LGG, Mitogen-Activated Protein Kinase (MAPK) pathway-altered [21].

In the era of targeted therapies, the identification of molecular alterations that are “druggable” are of utmost interest [19, 29, 34]. In this context, the discovery of *FGFR3::TACC3* fusion in a subset of tumors previously classified as glioblastoma [30] has prompted physicians to better characterize this group and to explore the therapeutic efficacy of FGFR inhibitors in patients with glioma harboring *FGFR3:TACC3* chromosomal translocation [13–15, 22, 31]. The molecular characterization of fusion-positive glioma revealed that *FGFR3::TACC3* is mutually exclusive with *IDH* and *H3* mutation but it is often associated with *TERT* promoter mutation and + 7/−10 chromosome copy-number changes and amplification of *CDK4* and /or *MDM2* [13, 31]. In adult setting, patients suffering from a glioblastoma, *IDH*-wildtype harboring *FGFR3::TACC3* fusions have a better survival rate than cases without *FGFR3::TACC3* fusion [13].

Interestingly, in the adult setting, diffuse gliomas with *FGFR3::TACC3* fusions have specific histopathological features that include oligodendrocyte-like cells, branched vessels, frequent calcifications and extravascular CD34 immunohistochemical expression [8]. Of interest, we and others have reported that these histopathological features are also shared by some pediatric-type diffuse LGG, i.e. PLNTY and a subgroup of diffuse LGG, MAPK pathway-altered [17, 23]. These two distinct tumor types might display *FGFR3::TACC3* fusion. However, fewer than 15 pediatric cases are reported in the literature and their prognosis is unknown, leading to difficulties in their clinical management [12, 17, 20, 23, 25, 28].

In the present study we report a multicenter series of adult and pediatric patients presenting with a diffuse oligodendroglioma-like glioma with a *FGFR3::TACC3* fusion, without (14 cases) or with (8 cases) features of microvascular proliferation and/or necrosis, in order to better characterize the clinico-pathological, molecular and epigenetic spectrum of these rare gliomas.

## Materials and methods

### Tumor samples

**Fig. 1:**
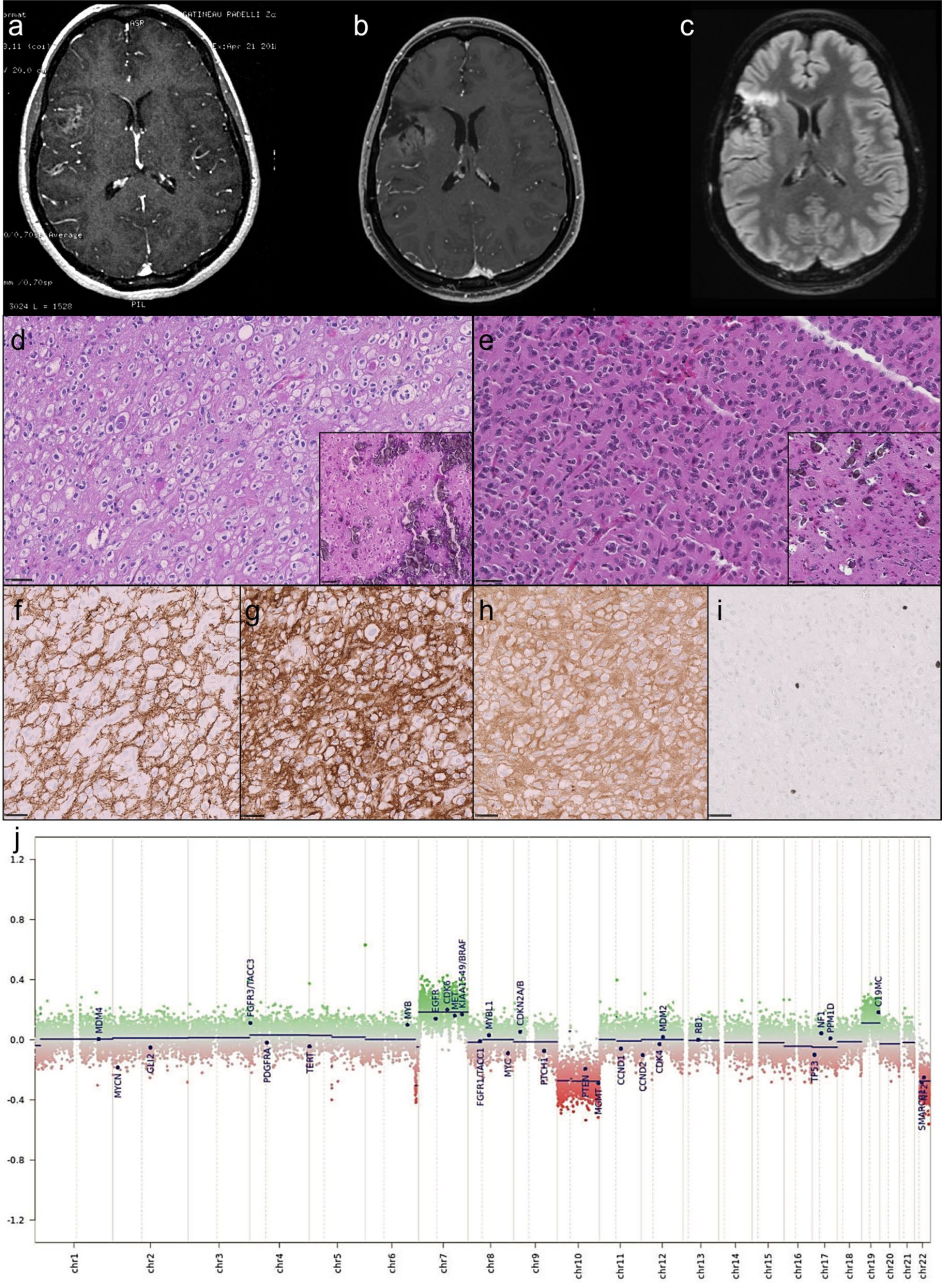
Illustration of an adult case presenting histologically as a low grade glioma (#12). This is an adult case of glioma (24 years at diagnosis) with FGFR3:TACC3 fusion with a low grade glioma (LGG) histological appearance initially and at recurrence. MRI at initial diagnosis showed a right frontal lesion with calcification and contrast enhancement (**a**). MRI at recurrence (**b**, **c**), showed an anterior high signal on FLAIR (**c**) without contrast enhancement (**b**). Histology at initial diagnosis (**d**, hematoxylin–phloxin–safron) and recurrence (**e** hematoxylin–phloxin–safron) showed an infiltrative and circumscribed growth pattern (**f** anti-neurofilamnet immunostaining), of an oligodendroglioma-like tumor with profuse microcalcifications (boxes) and without microvascular proliferation or tumoral necrosis. There was a strong and diffuse CD34 extra-vascular expression (**g** anti-CD34 immunostaining) and a strong and diffuse FGFR3 expression (**h** anti-FGFR3 immunostaining). No mitotic activity was observed and proliferation index was low (**i** anti-MIB1 immunostaining). Patient was simply monitored after surgeries and is still alive to date. Methylation class with v12.5 was ganglioglioma at both initial and recurrence with calibrated scores (CS) of respectively 0.41 and 0.61. Copy number variation (CNV) plots generated by the molecularneuropathology.org platform were the same between initial and recurrence (**j**). Gain of chromosome 7/loss of chromosome 10 was observed and there was mutation of TERT promotor. Scale bars: D, E: 50µm; F, G, H, I: 100 µm

The inclusion criteria were: diffuse oligodendroglioma-like gliomas, with a *FGFR3::TACC3* fusion detected by NGS using the FusionPlex® Lung kit by ArcherDX (ArcherDX Inc. Boulder, CO, USA), adapted to sequencing on MiSeq (Illumina Inc., San Diego, CA, USA), with or without features of microvascular proliferation and/or necrosis, and lacking *IDH1/2* or *H3* mutation tested by a brain tumor gene mutation panel developed using the MassARRAY iPlex technology and MassARRAY online design tools (Agena Bioscience) and with one available formalin-fixed-paraffin embedded (FFPE) block. A total of 22 cases fulfilled these criteria. The FFPE blocks concerned the first surgical excision in 20 cases, the initial excision (#12) and the recurrence (#12bis) in one (case #12, illustrated in Fig. 1) and the recurrence only in another one (case #21). These cases were retrieved from 8 University Hospital Centers: GHU Paris Psychiatry and Neurosciences, Marseille, Lille, Lyon, Toulouse, Nice, Montpellier and Rennes. Three cases (#13, #16 and #19) have been previously reported [2, 23]. Written informed consent to be included in this study was provided by the participants or participants’ legal guardian/next of kin. This study was reviewed and approved by the Aix-Marseille University ethics committee (2019-25-04-003). Clinical and radiological data were retrospectively collected for each case. Clinical data included sex, tumor location, age at diagnosis, type of resection and extent of surgical resection and follow-up (including date at last follow-up and date of progression or recurrence). Preoperative imaging (available for 21 patients) was collected and centrally reviewed by a senior neuroradiologist (MEG). Minimal imaging data consisted in at least the MRI report and if available, FLAIR and T1 with gadolinium injection MRI sequences. Well-defined ring-enhancement was assessed as present or absent to determine whether cases presented radiologically as high or low grade tumors in order to look for sampling issues in cases of glioblastoma, IDH wildtype as mentioned in Zhang et al. [27, 36].

### Pathological analysis and immunohistochemistry

Samples were stained with haematoxylin–phloxin–saffron (HPS) according to standard protocols. All tissue samples were centrally reviewed (DFB, AM). For each case, the following pathological features were searched for: diffuse or circumscribed growth pattern, microvascular proliferation, tumoral necrosis, calcification, dysmorphic ganglion cells, specific glioneuronal element, mitotic activity (/mm^2^). Search for immunohistochemical expression of OLIG2 (6F2, Dako®), CD34 (QBEnd10, Roche®), Neurofilament (2F11, Menarini®), Ki67 (MIB1, Dako®), FGFR3 (FGFR3, Clinisciences®) was performed. Cases presenting pathological features of microvascular proliferation and/or tumoral necrosis were classified as glioblastoma (GB), cases without microvascular proliferation and lack of tumoral necrosis were classified as low-grade glioma (LGG).

### DNA extraction and quantification

DNA was extracted from each tumor sample (n = 23) using the IDXTRACT-mag-FFPE kit (ID-Solutions, Grabels France) coupled to the automaton (IDEAL-32, ID-Solutions) as per the manufacturer’s directions. DNA was quality checked and quantified using DNA calibrated by an external standard range using the IDQUANTq kit (ID-Solutions) and the Mic® quantitative PCR instrument (Bio Molecular Systems, Queensland, Australia). Technical steps for DNA extraction were performed at La Timone University Hospital (Marseille). When insufficient DNA was extracted, additional sections were utilized.

### TERT promoter mutation

Mutation in *TERT* promoter was searched for by ddPCR assays according to previously published method [3].

### DNA methylation analysis

Samples containing at least 250 ng DNA were selected for DNA-methylation profiling (n = 23). DNA bisulfite conversion was undertaken using the ZymoEZ DNA methylation kit (Zymo research, USA), then treated with FFPE DNA Restore kit (Illumina, San Diego, CA, USA) and DNA clean and concentrator-5 (Zymo research, USA). Standard quality controls confirmed DNA quantity/quality and bisulfite conversion. The DNA was then processed using the Illumina Infinium HumanMethylation EPIC Bead-Chip array (Illumina, San Diego, CA, USA) according to the manufacturer’s instructions. The iScan control software was used to generate raw data files from the BeadChip in .idat format, analysed using GenomeStudio version 2.0 (Illumina, San Diego, CA, USA) and were checked for quality measures according to the manufacturer’s instructions.

### DNA methylation data processing

The .idat files were uploaded to the online CNS tumor DNA methylation classifier (11b4 and 12.5 versions) from the German Cancer Research Center (Deutsches Krebsforschungszentrum, DKFZ) at https://www.molecularneuropathology.org and a report for every tumor was generated, providing prediction scores for methylation classes (MC) and chromosomal copy-number-variation (CNV) plots. The scores were integrated to the histopathological findings according to the recommendations from Capper et al. [10, 11]. A prediction score > 0.9, in v12.5 or > 0.84 in v11b4, was considered high and relevant for diagnosis.

Additional analyses were performed in R studio (v4.0.2). Raw signal intensities were obtained from .idat files using the minfi Bioconductor package (v1.34.0). Background correction and dye-bias correction were performed on each sample. Filtering criteria of probes were removal of probes targeting X or Y chromosomes and removal of probes containing single nucleotide polymorphisms. Hierarchical clustering was performed using the Complex Heatmap package (2.4.3). Clustering of beta values from methylation arrays was performed based upon Euclidean distance with a ward algorithm. Methylation heatmaps show only the most variable probes (SD > 0.20). t-distributed stochastic neighbor embedding (t-SNE) dimensional reduction analysis was performed using the Rt-SNE package, with the following parameters: exaggeration factor = 42, normalize = TRUE, pca_scale = TRUE, pca_center = TRUE, eta = 500, theta = 0.01, max_iter = 5000, pca = TRUE. Visualization was done using ggplot2version 3.3.6 and Plotly version 3.10.0. In order to build the t-SNE we used as reference cohort cases retrieved from our DNA-methylation profiling database that were classified by the v12.5 version of the Heidelberg classifier with CS > 0.9 as follows: glioblastoma, *IDH*-wildtype mesenchymal subtype MC (12 cases); glioblastoma, *IDH*-wildtype RTK1 subtype MC (12 cases); glioblastoma, *IDH*-wildtype RTK2 subtype MC (12 cases); DNET MC (11 cases); and GG MC (10 cases).

### Chromosomal CNV analysis

Chromosomal CNV were searched for by visual inspection of CNV profiles generated by the *molecularneuropathology.org* platform as described [11, 32]. Visual inspection indicated: a gain of chromosome 7 if a complete gain was present; a deletion of chromosome 10 if a complete deletion was present; an *EGFR* amplification if a high-level amplification of *EGFR* locus was present.

### Statistical analysis

Categorical variables were presented as frequencies and continuous variables as medians and ranges. The Fisher exact test was used to compare categorical variables. Progression-free survival (PFS) was defined as the time from the date of diagnosis to recurrence or death from any cause. Overall survival (OS) was defined as the time from the date of diagnosis to death from any cause. The Kaplan–Meier method was used to estimate survival distributions. Log-rank tests were used for univariate comparisons. Statistical tests were two-sided, the threshold for statistical significance was *p* = 0.05. Statistical analysis was done using IBM SPSS Statistics software Version 27.0.0.0 (IBM, Bois-Colombes, France).

## Results

### Clinicopathological data

Ten women and 12 men (sex-ratio male/female 1.2) were included. The median age at diagnosis was 42.5 years (1–77). Six patients were aged less than 20 years (27%), 4 between 20 and 40 years (18%), 7 between 40 and 60 years (32%) and 5 above 60 years (23%). Fourteen cases had a history of epilepsy whereas six cases presented with a neurological deficit, and information was missing for 2 cases. Seventeen cases had undergone gross total resection and 4 had a biopsy or partial resection, and no information was available for the last case.

Follow-up data were available for all cases, with a median follow-up of 28.65 months (1.2–133.6 months). Twelve patients experienced recurrence or progression with a median time of 27.1 months (2–77.9 months). Five patients died from their disease with a median time of 15.6 months (11–28.3 months). Seven patients were treated according to Stupp protocol [33], 2 patients were treated with concomitant radiotherapy and chemotherapy, 3 patients received adjuvant anti-FGFR targeted therapy (cases #10 and #15 who died after respectively 11 and 28.3 months, and case #19 who was alive at last follow-up after 37.3 months post-surgery).

Nineteen tumors were supratentorial (7 temporal, 6 frontal, 3 parietal, 3 in more than one lobe but always involving the temporal lobe) whereas in 3 cases the location was infratentorial and median (2 in the fourth ventricle and one in the mesencephalon). Altogether 10 cases involved the temporal lobe and 12 did not. Ten cases presented radiologically with well-defined ring-enhancement suggestive of neoangiogenesis and a high-grade tumor (Table 1). Eleven cases did not have contrast enhancement. Pre-operative imaging was not available in one case.

**Table 1:** Molecular characteristics

No	Age at initial diagnosis	Histological diagnosis	Contrast enhancement	Fusion	TERT promoter	Chr + 7/− 10	WHO 2021	Hierarchical clustering	t-SNE cluster	MC v11b4	CS v11	MC v12.5	CS v12.5
#01	56	GB	Present	FGFR3(ex18)::TACC3(int6)	Mutated	Present	GB IDH-WT	Group 2	GB	Glioblastoma IDH wildtype	0.99	GB_RTK2	0.94
#02	44	GB	Present	FGFR3(ex17)::TACC3(ex11)	Mutated	Present	GB IDH-WT	Group 2	GB	Glioblastoma IDH wildtype	0.99	GB_RTK2	0.57
#03	65	LGG	Present	FGFR3(ex17)::TACC3(ex4)	Mutated	Present	GB IDH-WT	Group 2	GB	Glioblastoma IDH wildtype	0.99	GB_MES_TYP	0.65
#04	63	GB	Present	FGFR3(ex17)::TACC3(ex10)	Mutated	Present	GB IDH-WT	Group 2	GB	Glioblastoma IDH wildtype	0.99	GB_MES_TYP	0.97
#05	58	GB	Present	FGFR3(ex17)::TACC3(ex8)	Mutated	Present	GB IDH-WT	Group 4	GB	Glioblastoma IDH wildtype	0.99	GG	0.36
#06	68	GB	Present	FGFR3(ex17)::TACC3(ex11)	Mutated	Present	GB IDH-WT	Group 3	GB	Glioblastoma IDH wildtype	0.71	GB_MES_TYP	0.81
#07	56	LGG	No	FGFR3(ex17)::TACC3(ex11)	Mutated	Absent	GB IDH-WT	Group 4	GB	Glioblastoma IDH wildtype	0.90	GG	0.54
#08	60	GB	Present	FGFR3(ex17)::TACC3(ex11)	Mutated	Present	GB IDH-WT	Group 3	GB	Glioblastoma IDH wildtype	0.85	GB_MES_TYP	0.89
#09	77	LGG	No	FGFR3(ex17)::TACC3(ex10)	Mutated	Present	GB IDH-WT	Group 3	GB	Glioblastoma IDH wildtype	0.67	GB_MES_TYP	0.84
#10	45	LGG	NA	FGFR3(ex17)::TACC3(ex11)	Mutated	Absent	GB IDH-WT	Group 3	GB	plexus tumor	0.39	GB_MES_TYP	0.31
#11	41	GB	Present	FGFR3(ex17)::TACC3(ex11)	Mutated	Present	GB IDH-WT	Group 4	GB	NM	< 0.3	GG	0.23
#12	24	LGG	No	FGFR3(ex17)::TACC3(ex10)	Mutated	Present	GB IDH-WT	Group 4	GB	LGG, GG	0.13	GG	0.41
#12bis	NA	LGG	NA	FGFR3(ex17)::TACC3(ex10)	Mutated	Present	GB IDH-WT	Group 4	GB	Glioblastoma IDH wildtype	0.74	GG	0.61
#13	4	LGG	Present	FGFR3(ex17)::TACC3(ex10)	Wildtype	Absent	PLGG	Group 3	GG	NM	< 0.3	IHG	0.26
#14	12	LGG	No	FGFR3(ex17)::TACC3(ex13)	Wildtype	Absent	PLGG	Group 3	GG	LGG, GG	0.99	GG	0.99
#15	72	LGG	Present	FGFR3(ex17)::TACC3(ex11)	Mutated	Absent	GB IDH-WT	Group 3	GG	LGG, GG	0.70	GG	0.99
#16	10	LGG	No	FGFR3(ex17)::TACC3(ex12)	Wildtype	Absent	PLGG	Group 3	GG	LGG, GG	0.89	GG	0.99
#17	38	LGG	No	FGFR3(ex18)::TACC3(ex10)	Mutated	Present	GB IDH-WT	Group 3	GG	LGG, GG	0.59	GG	0.98
#18	13	LGG	No	FGFR3(ex17)::TACC3(ex10)	Wildtype	Absent	PLGG	Group 1	DNET	LGG, DNET	0.6	DNET	0.77
#19	29	LGG	No	FGFR3(ex17)::TACC3(ex10)	Wildtype	Absent	PLGG	Group 1	DNET	LGG, DNET	0.26	RGNT	0.28
#20	6	LGG	No	FGFR3(ex17)::TACC3(ex10)	Wildtype	Absent	PLGG	Group 1	DNET	LGG, DNET	0.99	DNET	0.98
#21bis	29	LGG	No	FGFR3(ex17)::TACC3(ex10)	Wildtype	Absent	PLGG	Group 1	DNET	LGG, DNET	0.56	MYXGNT, PDGFRA-mutant (novel)	0.57
#22	1	LGG	No	FGFR3(ex17)::TACC3(ex10)	Wildtype	Absent	PLGG	Group 1	DNET	LGG, DNET	0.48	GG	0.39

*CS* calibrated score, *DNET* dysembryoplastic neuroepithelial tumor methylation class, *Ex* exon, *GB* glioblastoma methylation class by t-SNE clustering, *GB IDH-WT* glioblastoma, *IDH*-wildtype integrated diagnosis according to 2021 World Health Organization classification, *GB* glioblastoma at histology, *GB_MES_TYP* glioblastoma mesenchymal subtype methylation class, *GB_RTK2* glioblastoma RTK2 subtype methylation class, *GG* ganglioglioma methylation class, *IHG* infantile hemispheric glioma methylation class, *In* intron, *LGG* low grade glioma at histology, *LGG, DNET* low grade glioma dysembryoplastic neuroepithelial tumor methylation class, *LGG, GG* low grade glioma ganglioglioma methylation class, *MC* methylation class, *LGG* low grade glioma histology, *MYXGNT, PDGFRA-mutant (novel)* myxoid glioneuronal tumor PDGFRA-mutant methylation class (novel), *NA* not available, *NM* no match, *LGG* pediatric low grade glioma integrated diagnosis according to 2021 World Health Organization classification, *RGNT* rosette forming glioneuronal tumor methylation class

**Fig. 2:**
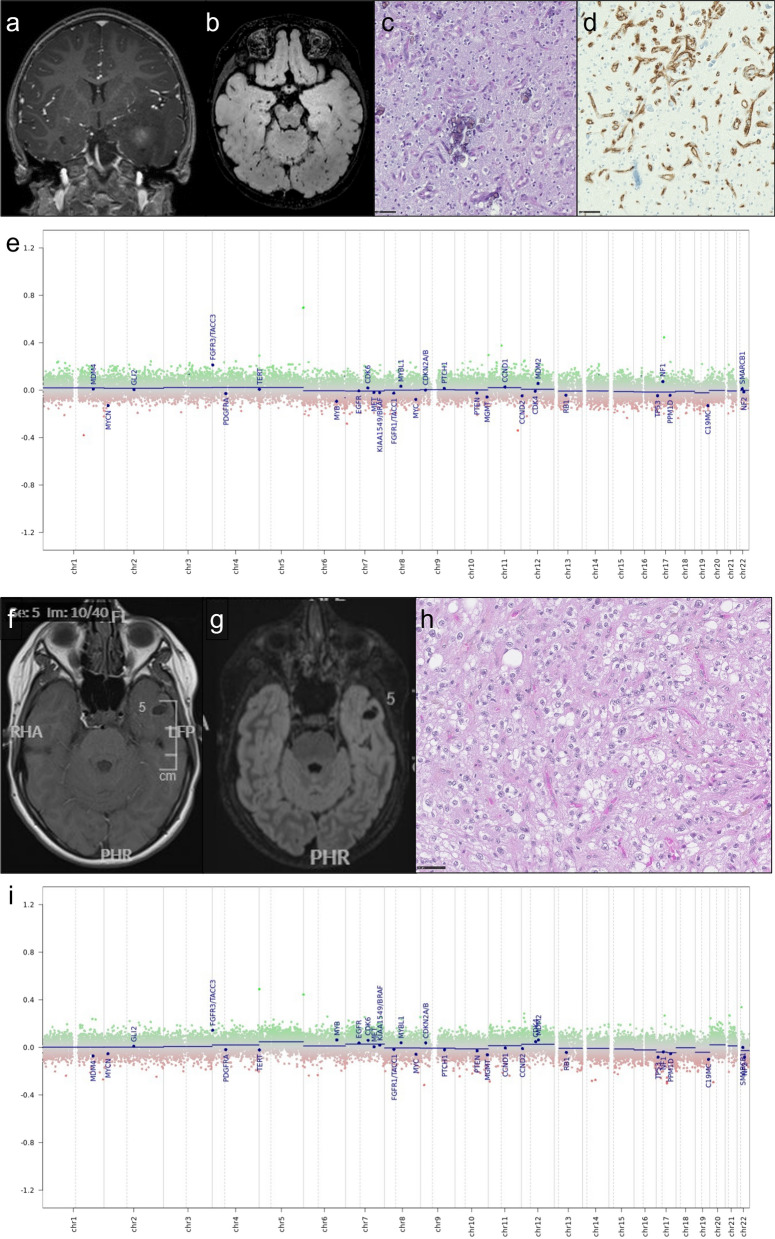
Illustration of a pediatric case of FGFR3::TACC3 low grade glioma (#22 & #14). Upper panel **a**–**e** illustrates the case of a 1-year-old at diagnosis child (#22). Clinical presentation was a West syndrome starting at 6 months of life. MRI revealed a left temporal infiltrative tumor (**a** T1 with gadolinium injection; **b** FLAIR sequence). Surgical resection was performed at 1 year old. Histological examination **c** showed a diffuse glial tumor with calcifications, there was no microvascular proliferation, no necrosis, nor mitotic activity. There was no CD34 extravascular expression (**d**). Methylation class with v12.5 was ganglioglioma with a calibrated score of 0.39, and the case clustered with ganglioglioma cluster on t-SNE, it fell within cluster 1 with other low-grade tumors on hierarchical clustering. Copy number plot generated by the platform did not show copy number variation (**e**). Lower panel **f**–**i** illustrates the case of a 12-year-old child (#14) with chronic epilepsy. MRI show a left temporal without contrast enhancement (**f** T1 with gadolinium injection, **g** FLAIR sequence), surgical resection at 12 years old and revealed a diffuse oligodendroglioma-like tumor with calcification and strong and diffuse CD34 expression and FGFR3(ex17)::TACC3(ex13) fusion, very suggestive of a polymorphous low grade neuroepithelial tumor of the young (PLNTY) in the absence of ganglion cells (**h** hematoxylin–phloxin–saffron). There was no TERT promoter mutation and no chromosome + 7/− 10 (**i** CNV plots from DNA methylation profiling). Methylation class was ganglioglioma with 0.99 calibrated score. Scale bars: 50 µm

Among the 22 cases reviewed, 14 presented histopathological features of LGG (no microvascular proliferation, no necrosis and lack of mitotic activity, Fig. 2) and 8 had histopathological features of glioblastoma (microvascular proliferation and/or necrosis). Except one case which was extensively calcified, all tumors demonstrated a diffuse growth pattern on anti-neurofilament immunostaining. Calcifications were recorded in 16/21 cases (10 LGG cases and 6 GB), and information was lacking in one. Specific glioneuronal element and dysmorphic ganglion cells were not observed. All cases displayed a strong and diffuse immunohistochemical expression of FGFR3. Extravascular CD34 expression was observed in 14/21 cases (10 LGG cases and 4 GB), and not available in one.

### Molecular data

*TERT* promoter mutation was recorded in 14 cases, absent in 8. Importantly all cases with histopathological features of glioblastomas displayed *TERT* promoter mutation but also 6/14 with histopathological features of LGG cases. Chromosomes + 7/−10 was observed in 11 cases including the 8 cases with pathological features of glioblastoma and 3 LGG cases and was always associated with *TERT* promoter mutation. *EGFR* amplification was never detected. Molecular data are summarized in Table 1. The most frequent fusion types were *FGFR3*(Ex17)::*TACC3*(Ex10) (9/22 cases) and *FGFR3*(Ex17)::*TACC3*(Ex11) (7/22 cases).

### DNA methylation profiling data

For each case, the DNA MC and the CS according to the v11b4 and the v12.5 versions of the DKFZ classifier are summarized in Table 1. Seven samples had a CS above 0.9 with v12.5 for the following MC: ganglioglioma (4 cases), glioblastoma, *IDH*-wildtype, RTK2 or mesenchymal subtypes (2 cases), dysembryoplastic neuroepithelial tumor (1 case). Fifteen cases had a CS under 0.9 with v12.5 for the following MC: glioblastoma, *IDH*-wildtype, RTK2 or mesenchymal subtypes (6 cases), ganglioglioma (5 cases), dysembryoplastic neuroepithelial tumor (1 case), infantile hemispheric glioma (1 case), rosette forming glioneuronal tumor (1 case), myxoid glioneuronal tumor, *PDGFRA*-mutant (novel) (1 case). For the only case (#12) that benefited from two DNA methylation analyses (performed on the initial and recurrence surgeries), both specimens were classified as GG MC with the v12.5, although the CS were low (0.41 and 0.60). Interestingly the recurrence sample (#12bis) was classified by v11b4 in the glioblastoma *IDH*-wildtype MC family (mesenchymal subtype) with a low score and reclassified as GG by v12.5 (Fig. 1). Moreover, 2 cases (#05 and #07) were classified as glioblastoma, *IDH*-wildtype (mesenchymal subtype) in v11b4, and reclassified as GG (with low CS, respectively 0.36 and 0.54) with the v12.5 version of the classifier. However, because of their low confidence score, these cases should be considered as “non classifiable” especially because the DNA MC changed from one version of the classifier to another and therefore emphasizing that the information given by the DNA methylation analysis might be questionable in some cases. Importantly, 2 cases (#15 and #17) classified as LGG, GG by v11b4 with CS < 0.84 received a higher score for the same MC with v12.5 (respectively 0.99 and 0.98). None received a MC of PLNTY.

### T-distributed stochastic neighbor embedding analysis of DNA methylation data

**Fig. 3:**
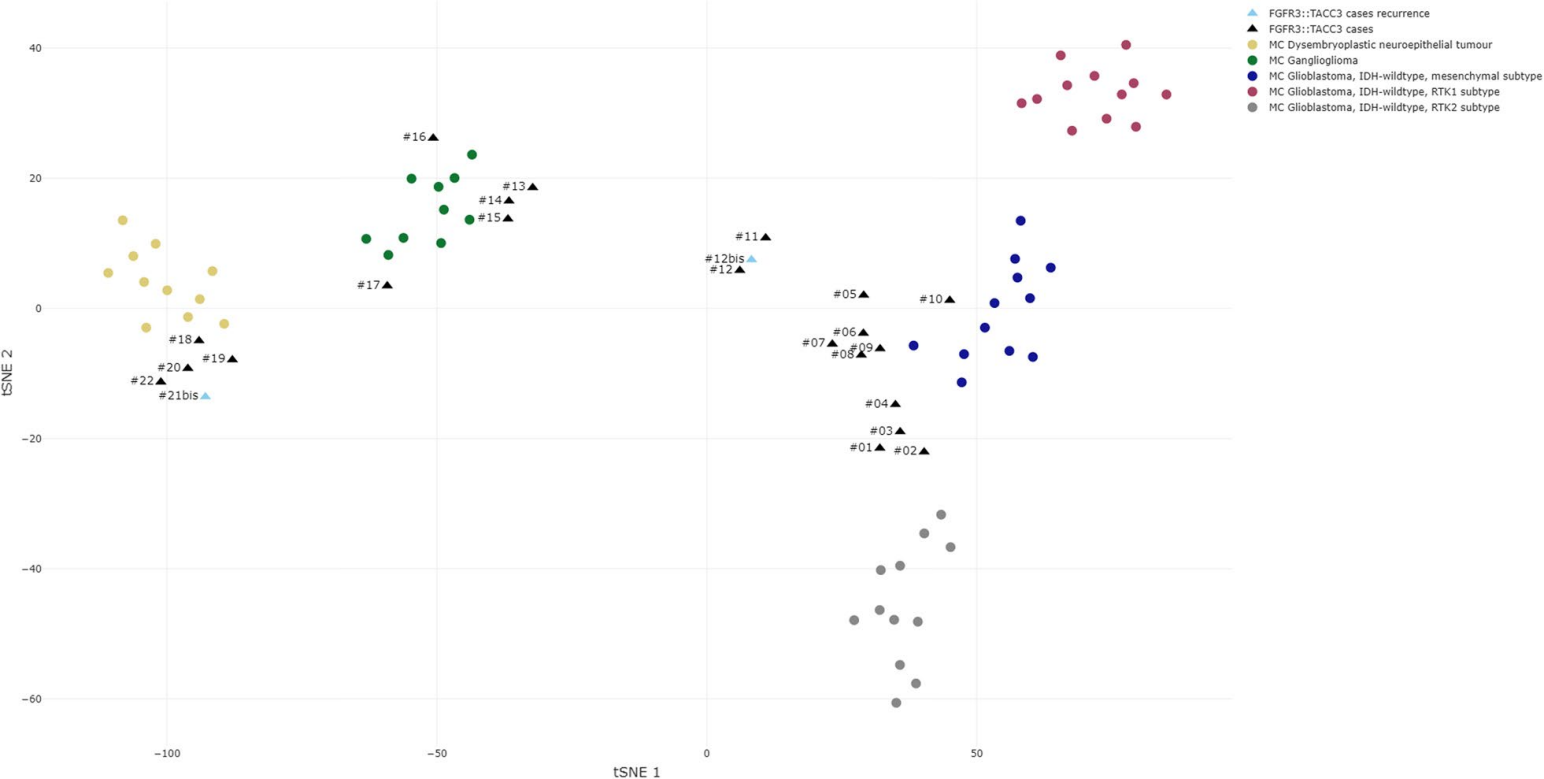
t-distributed stochastic neighbor embedding DNA-methylation profiling data analysis. t-SNE analysis of 23 glioma samples with FGFR3::TACC3 fusion (black triangles and blue triangles for recurrences) compared to reference cohort samples retrieved from our DNA-methylation profiling database that were classified by the v12.5 version of the Heidelberg classifier with calibrated scores (CS) > 0.9 as follows: glioblastoma, IDH-wildtype mesenchymal subtype methylation class (MC) (12 cases, blue dots); glioblastoma, IDH-wildtype RTK1 subtype MC (12 cases, purple dots); glioblastoma, IDH-wildtype RTK2 subtype MC (12 cases, grey dots); LGG DNET MC (11 cases, yellow dots); and LGG GG MC (10 cases, green dots)

t-SNE analysis showed that the 22 cases clustered into three main groups (Fig. 3). The first one, close to glioblastoma, *IDH*-wildtype MC (mesenchymal and RTK2 subtypes), formed a separate group of 12 cases (cases #1 to #12 and its relapse, #12bis) and was therefore named glioblastoma or GB cluster. The second group of 5 cases clustered tightly with ganglioglioma MC (cases #13 to #17) and was therefore named ganglioglioma or GG cluster. The third group of 5 cases formed a separate cluster in close vicinity of dysembryoplastic neuroepithelial tumor MC (cases #18 to #22), and was therefore named dysembryoplastic neuroepithelial or DNET cluster. Therefore, 10 cases had a DNA-methylation profile close to pediatric low grade glioneuronal tumors whereas 12 were closer to glioblastoma, *IDH*-wildtype.

### Unsupervised clustering analysis based on DNA-methylation data

**Fig. 4:**
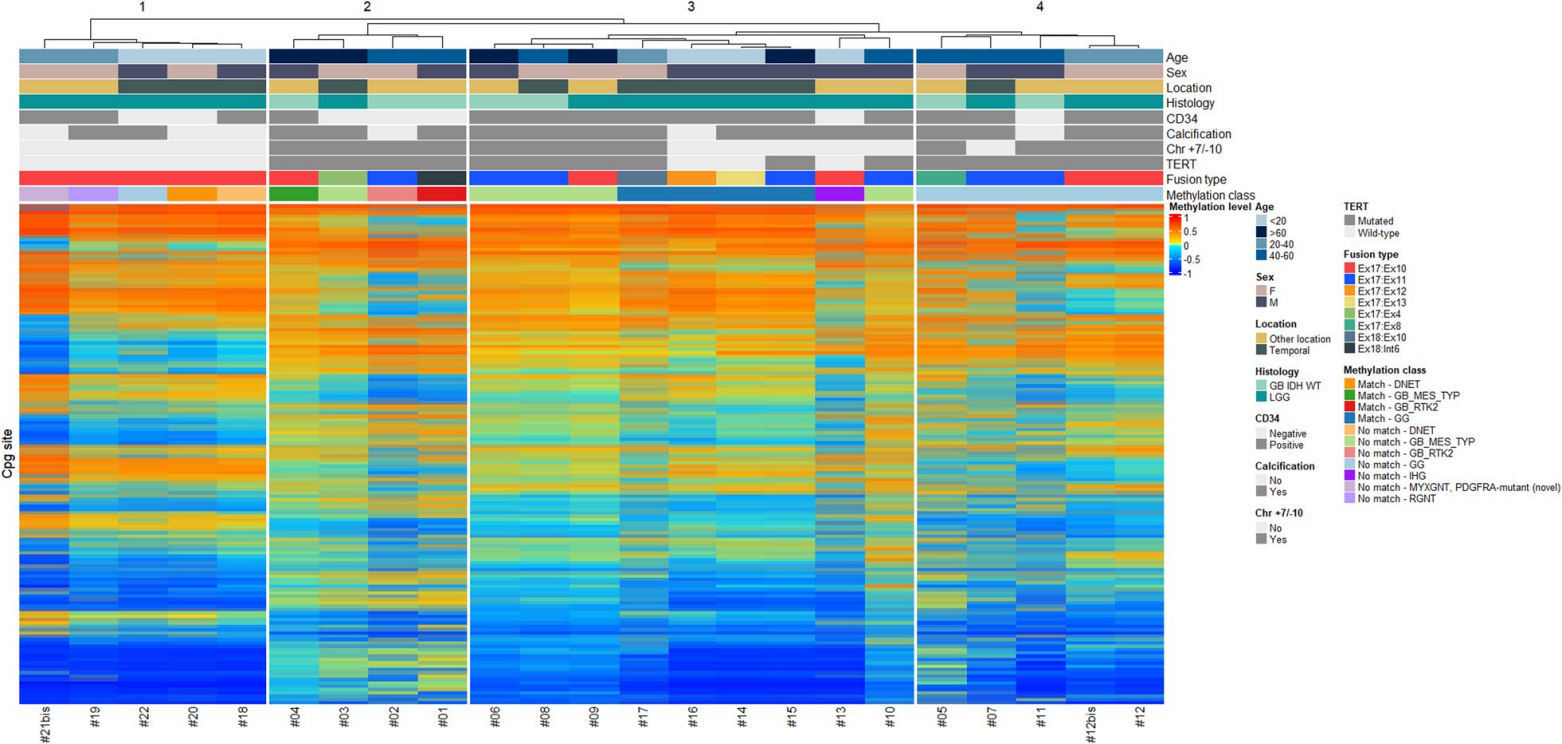
Unsupervised hierarchical clustering of DNA-methylation profiling data. Unsupervised hierarchical clustering of 23 glioma samples with FGFR3:TACC3 fusion based on the 10,000 most variably methylated probes. Samples with CS > 0.9 were considered as matching with the MC proposed by the v12.5 version of the DKFZ classifier, CS ≤ 0.9 were considered not matching. *Chr* chromosome, *DNET* dysembryoplastic neuroepithelial tumor, *GG* ganglioglioma, *IHG* infantile hemispheric glioma, *LGG* low grade glioma, *GB IDH-WT* glioblastoma *IDH*-wildtype, *GB_MES_TYP* glioblastoma mesenchymal subtype, *GB_RTK2* glioblastoma RTK2 subtype, *MYXGNT,* *PDGFRA-mutant (novel)* Myxoid glioneuronal tumor, PDGFRA-mutant (novel), *RGNT* rosette forming glioneuronal tumor

Unsupervised hierarchical clustering based on our series of 22 cases classified at histology as GB (8 cases) or LGG (14 cases) revealed four main groups (1–4) although group 1 was clearly distinct from the three others (Fig. 4). Group 1 comprised 5 cases (cases #18 to 22) that shared young age (< 40 years), pathological features of LGG, lack of *TERT* promoter mutation, lack of chromosome + 7/− 10, *FGFR3*(Exon 17)::*TACC3*(Exon 10) fusion type and displayed a MC of LGG. Group 2 was made up of 4 cases (cases #1 to #4) that shared old age (> 40 years), pathological features of glioblastoma, presence of *TERT* promoter mutation, and chromosome + 7/−10. Group 3 comprised 9 cases (cases #6, #8, #9, #10 and #13 to 17) and group 4 comprised 4 cases (cases #5, #7, #11, #12 and its relapse, #12bis), and were more heterogeneous. Five cases from group 3 displayed a MC of high-grade glioma (4 glioblastomas, *IDH*-wildtype MC and one infantile hemispheric glioma MC) and 4 others had a MC of LGG GG. Two of nine cases had histopathological features of glioblastoma, *IDH*-wildtype, whereas the 7/9 other cases demonstrated pathological features of LGG but among them 4 demonstrated *TERT* promoter mutation associated in two cases with chromosome + 7/− 10. Interestingly, the 3/7 cases that had features of LGG but no *TERT* promoter mutation were less than 20 years old. In contrast, all cases of group 4 displayed a MC of GG and *TERT* promoter mutation although histopathological features were LGG in two cases and GB in the two others. None of these patients were younger than 20 years nor older than 60 years.

### Correlation with clinical, radiological and molecular data

Although some correlations reached significance, Fisher’s test results should be viewed with caution given the small number of subjects (Additional file 1: Table S1). Age at diagnosis under 40 years was significantly associated with LGG histology at initial diagnosis (*p* = 0.014). *TERT* promoter mutation was significantly more frequent in older patients (> 40 at initial diagnosis) (*p* = 0.001). *TERT* promoter mutation was significantly associated with GB cluster (*p* < 0.0001) and *FGFR3*(Ex17)::*TACC3*(Ex11) fusion type (*p* = 0.022). Younger patients (< 40 years) were more likely to fall within the GG/DNT cluster (*p* = 0.002), whereas older patients (> 40 years) were more likely to fall within the GB cluster. Interestingly, tumors of GB cluster were more likely to develop outside the temporal lobe, whereas the tumors of GG/DNT cluster were mainly located in the temporal lobe (*p* = 0.017). Importantly, some cases that had pathological features of LGG and belong to GB cluster after t-SNE analysis were initially classified as GG MC with the v12.5 version of the classifier. This is the case for case #12 and its relapse, #12bis, suggesting that the MC per se is not predictive of the prognosis especially if the score is low.

Besides, two LGG (cases #13 and #15) had well-defined contrast enhancement suggestive of a surgical “undersampling”. However, both cases clustered in GG/DNT cluster in t-SNE and did not have chromosome + 7/− 10, although case #15 (72 year-old) presented a *TERT* promoter mutation with a score of 0.99 for GG MC in v12.5. This last case had a progression at 28 months and died of his disease after 66 months. Again, this further highlights that DNA methylation profiling is neither a grading tool nor a substitute for histological diagnosis and when the DNA MC class is discrepant with pathological features, caution must be paid, even if the score is high. All histological glioblastomas had imaging features of high grade gliomas.

### Search for prognostic factors

#### Survival analysis according to age at first diagnosis

**Fig. 5:**
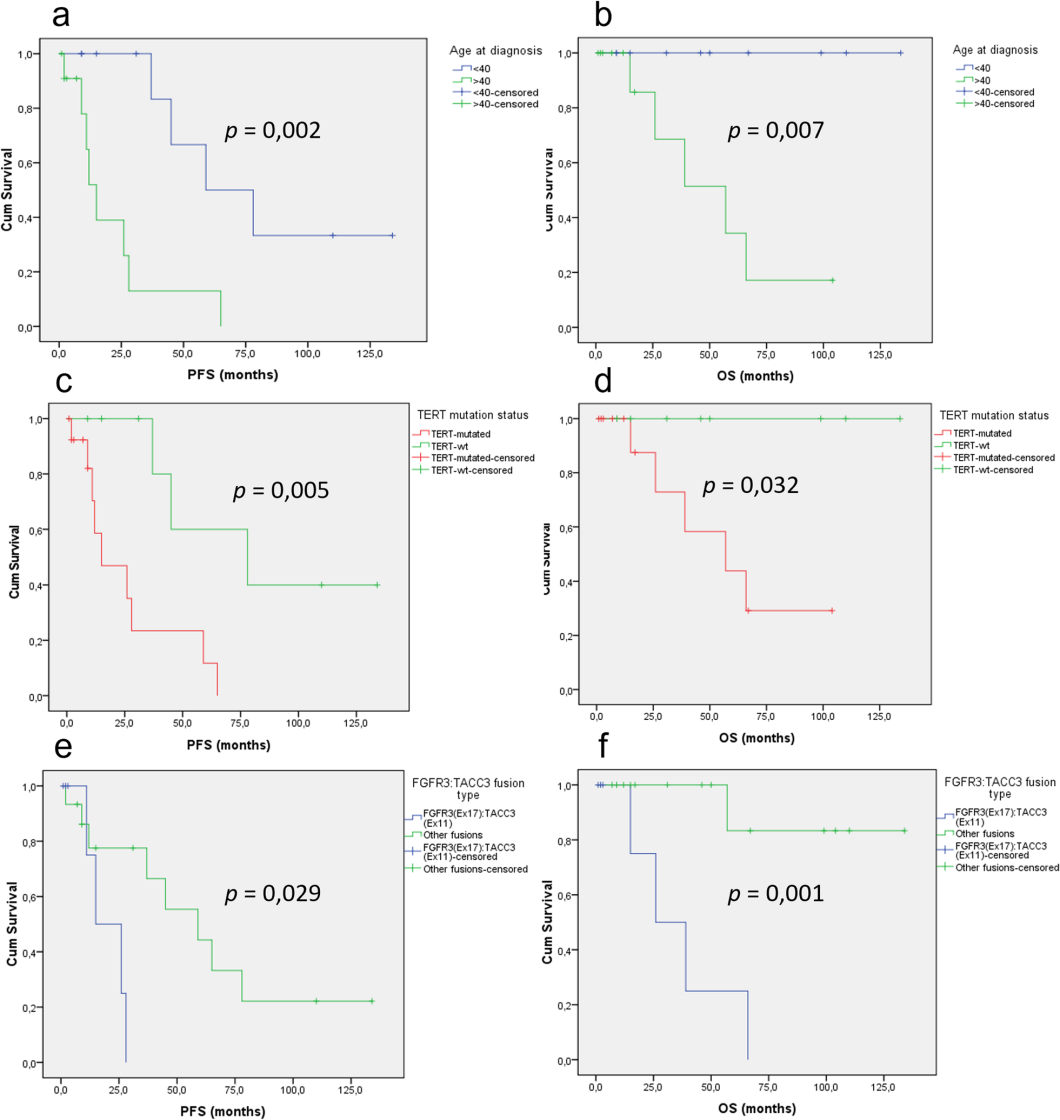
Survival analysis according to WHO CNS 5 diagnosis and age at diagnosis. Kaplan-Meier curves for progression-free (**a**) and overall (**b**) survival for gliomas with *FGFR3:TACC3* fusion according to age at initial diagnosis (> 40 vs. < 40). Kaplan–Meier curves for progression-free (**c**) and overall (**d**) survival for gliomas with *FGFR3:TACC3* fusion according to TERT promotor mutation status (mutated vs. wild-type). Kaplan–Meier curves for progression-free (**e**) and overall (**f**) survival for gliomas with *FGFR3:TACC3* fusion according to *FGFR3:TACC3* fusion type (*FGFR3*(Ex17)::*TACC3*(Ex11) vs. other fusions). *OS* overall survival, *PFS* progression-free survival

 Patients under 40 years of age have a significantly better PFS and OS than patients over 40 years of age (respectively *p* = 0.002 and *p* = 0.007) (Fig. 5a, b).

#### Survival analysis according to tumor location

There was no statistically significant difference between PFS (*p* = 0.137) and OS (*p* = 0.299) for tumors located in the temporal lobe (or involving the temporal) and the non-temporal tumors.

#### Survival analysis according to histological diagnosis

Patients with pathological features of glioblastoma had a worse PFS (*p* = 0.012) than LGG but OS was not significantly different (*p* = 0.065).

#### Survival analysis according to TERT promoter mutation

Whatever the histological diagnosis or the t-SNE clustering, it was obvious that cases with *TERT* promoter mutation had a worse prognosis in comparison with cases lacking *TERT* promoter mutation for both PFS (*p* = 0.005) and OS (*p* = 0.032) (Fig. 5c, d).

#### Survival analysis according to fusion type

In our series of diffuse gliomas with *FGFR3*::*TACC3* fusion, PFS (*p* = 0.029) and OS (*p* = 0.001) were significantly worse in gliomas demonstrating *FGFR3*(Ex17)::*TACC3*(Ex11) fusion (Fig. 5e, f). In contrast, gliomas with *FGFR3*(Ex17)::*TACC3(Ex10)* fusion had a significantly better OS (*p* = 0.032) but PFS was not significantly different (*p* = 0.328).

#### Survival analysis according to t-SNE clustering

Patients from GG/DNT cluster had a significantly better PFS than patients from GB cluster (*p* = 0.024) but OS was not significantly different (*p* = 0.126).

## Discussion

Diffuse gliomas with *FGFR3::TACC3* fusion have been mainly reported in the adult setting where they account for 3.1–11.8% of cases with pathological features of glioblastoma [4, 13, 22, 30, 31]. The frequency of *FGFR3::TACC3* fusion in gliomas with pathological features of low grade is more difficult to appreciate but does not exceed 4% in some studies [16, 23, 31]. Importantly, fewer than 15 *FGFR3::TACC3* pediatric gliomas, presenting initially with LGG histological features, have been reported to date, including one case of PLNTY with secondary malignant transformation [1, 6, 12, 17, 18, 20, 23, 25, 28].

### Diffuse gliomas with *FGFR3::TACC3* fusion display some recurrent pathological features but heterogeneous epigenetic signature and clinical behavior

Whatever the age of patients and location, diffuse gliomas with *FGFR3::TACC3* fusion display recurrent pathological features including oligodendroglioma-like cells, branched vessels, often associated with calcifications and CD34 extra-vascular expression associated in some cases with pathological features of glioblastoma (microvascular proliferation and/or necrosis) [6–9, 12, 17, 20, 23, 26].

In contrast, diffuse gliomas with *FGFR3::TACC3* fusion display epigenetic heterogeneity and do not form a single MC cluster. Glioblastomas with *FGFR3::TACC3* fusion are mainly distributed between mesenchymal and RTK2 MC subtypes [22]. Consistently, most of the cases of the present study formed a quite homogeneous cluster on t-SNE, close to GBM-mesenchymal subtype MC and GBM RTK2 subtype MC but far away from the GBM RTK1 subtype MC, the other cases belonging to LGG clusters. Interestingly, a previous study identified among 79 glioblastomas with *FGFR3::TACC3* fusion 11 cases that formed on t-SNE analysis a separate cluster (named ‘outlier’ *FGFR3::TACC3* positive glioblastoma, GBM-F3T3-O) close to the cluster of GBM-mesenchymal subtype [35]. Among them, 5/11 cases had pathological features of glioblastoma, and 6 had features of LGG. Although these cases were originally classified as GBM-mesenchymal subtype by the v11b4 version of the classifier, 7/11 were reclassified as LGG GG with the v12.5 version, but the CS were low. Accordingly, none of these cases were found in the ganglioglioma cluster in the t-SNE performed by the authors. In the present study we also recorded cases whose MC scores changed between v11b4 and v12.5: from glioblastoma, *IDH*-wildtype (mesenchymal subtype) MC to GG MC (v12.5 version) or LGG MC in v12.5 and indeed clustered in DNT or GG cluster by t-SNE analysis; but we also observed cases that clustered with GB by t-SNE analysis. Altogether these results suggest that t-SNE analysis might provide important information when the score for a MC remains low.

In addition to the epigenetic heterogeneity, we also observed that the clinical behavior of glioma with *FGFR3::TACC3* fusion was also heterogeneous with some patients that had a stable disease in contrast to others.

### The importance of age, epigenetic signature, TERT promoter mutation and FGFR3::TACC3 fusion subtype for prognostic stratification

Although our study comprised a limited number of cases, it highlights that patients younger than 40 years with pathological features of LGG, lack of *TERT* promoter mutation and *FGFR3(Ex17)::TACC3(Ex10)* fusion have a good prognosis in contrast to those that are older than 40 and display *TERT* promoter and *FGFR3(Ex17)::TACC3(Ex11)* fusion. Although DNA methylation profiling may suggest a prognostication by an epigenetic signature of LGG or a matching methylation class associated to a low-grade tumor set, it must be remembered that classification using methylation profiling remains a tool for research use and does not predict the individual prognosis of the case tested and therefore cannot be considered as a grading tool. Indeed, the case #15 in the present study had progression and died after 66 months despite having an histology of LGG with a matching methylation class of GG (score 0.99) and clustering by t-SNE analysis with GG.

Further studies are required to confirm these new findings.

### Diffuse gliomas with FGFR3::TACC3 fusion: a nosological issue

Diffuse gliomas with *FGFR3::TACC3* fusion are not recognized as a distinct tumor type in the 2021 WHO classification of CNS tumors, which is in keeping with their clinical and epigenetic heterogeneity. This leads to major difficulties in their subtyping and grading according to the WHO 2021 [21]. In fact, when these diffuse gliomas occur in adults, it is easy to classify them among the group of glioblastoma, *IDH*-wildtype grade 4 if the tumors display pathological or molecular features of glioblastomas [21]. As relevantly reported, such cases might also represent two subgroups of glioblastomas: early/evolving or “undersampled” depending on the absence or presence of typical neuroradiological features of glioblastoma, respectively [13, 22]. But we have to keep in mind that among glioblastoma, *IDH*-wildtype, those that harbor *FGFR3::TACC3* fusion display a better prognosis than the others [13, 22].

In contrast, in pediatric setting and in young adults, the finding of a *FGFR3::TACC3* fusion in a tumor displaying pathological features of LGG (whatever the neuroradiological findings) leads to major difficulties in its classification. In the absence of DNA methylation profiling, the pathologist can choose between PLNTY (grade 1) or diffuse LGG, MAPK-pathway-altered (no grade attributed yet). In fact, the diagnosis of PLNTY relies on the following essential criteria: “diffuse growth pattern and oligodendroglioma-like component and few (if any) mitotic figures and regional CD34 expression and IDH-wild type status and BRAF V600E mutation or FGFR2 or 3 fusions”. Eight cases (#09, #12, #14, #16–19, #21) of the present series fulfilled these criteria although for none of them was proposed a PLNTY MC by the v12.5 of the DKFZ classifier (MC were: GG, DNET, GB_MES_TYP, RGNT, MYXGNT, PDGFRA-mutant (novel)). Of interest, the only case with *FGFR3::TACC3* fusion included in the first description of PLNTY was not analyzed by DNA methylation [17]. Therefore, caution should be used when diagnosing PLNTY in patients with LGG and *FGFR3::TACC3* fusion, especially because malignant transformation associated with *TERT* promoter mutation at recurrence has been reported [6, 7]. The other alternative diagnosis: “diffuse LGG, MAPK-pathway-altered” might be preferred although this tumor type lacks specific pathological features as well as epigenetic signature [5, 23]. Besides, the prognostic significance of a *TERT* promoter mutation in these two tumor types, PLNTY and diffuse LGG, MAPK-pathway-altered, has not been fully elucidated yet as well, and there are yet no guidelines to classify such tumors. As shown by the present study, alternative diagnoses such as DNET and GG can be proposed by DNA methylation profiling with high confidence score, however this proposition should be taken with caution in the absence of characteristic pathological features (glioneuronal element, neoplastic ganglion cells). To date, histology takes precedence over the methylation class in these two tumor type classifications according to WHO CNS5 2021. To the best of our knowledge, except for one case of the present series, *FGFR3::TACC3* fusion has never been reported in cases classified by DNA methylation profiling as DNET, even in large series [24]. Besides, the prognostic significance of *TERT* promoter mutation in cases with a methylation score of ganglioglioma is largely unknown.

To conclude, although glioma with *FGFR3::TACC3* fusion is not a distinct tumor type in the WHO 2021 classification of CNS tumor, searching for *FGFR3::TACC3* fusion in diffuse *IDH*-wildtype glioma especially if it displays an oligo-like appearance and whatever the age of the patient is of utmost importance since these tumors might benefit from targeted therapy, although the evidence for their efficacy remains limited to date.

Age, *TERT* promoter mutation, combined pathological features, DNA-methylation profiling as well as fusion subtype are of interest to determine patients’ stratification risk.

## Supplementary Information

**Additional file 1: Table S1.** Correlation table (Fisher’s exact tests).

## Data Availability

All data generated or analysed during this study are included in this published article.

## References

[1] Ahrendsen JT, Sinai C, Meredith DM, Malinowski SW, Cooney TM, Bandopadhayay P, Ligon KL, Alexandrescu S (2021). Molecular alterations in Pediatric Low-Grade Gliomas that led to death. J Neuropathol Exp Neurol.

[2] Appay R, Bielle F, Sievers P, Barets D, Fina F, Boutonnat J, Adam C, Gauchotte G, Godfraind C, Lhermitte B, Maurage C-A, Meyronet D, Mokhtari K, Rousseau A, Tauziède-Espariat A, Tortel M-C, Uro-Coste E, Burel-Vandenbos F, Chotard G, Pesce F, Varlet P, Colin C, Figarella-Branger D (2022). Rosette-forming glioneuronal tumours are midline, FGFR1-mutated tumours. Neuropathol Appl Neurobiol.

[3] Appay R, Fina F, Barets D, Gallardo C, Nanni-Metellus I, Scavarda D, Henaff D, Vincent J, Grewis L, Pourquier P, Colin C, Figarella-Branger D (2020). Multiplexed droplet digital PCR assays for the simultaneous screening of major genetic alterations in tumors of the central nervous system. Front Oncol.

[4] Asif S, Fatima R, Krc R, Bennett J, Raza S (2019). Comparative proteogenomic characterization of glioblastoma. CNS Oncol.

[5] Bale TA, Rosenblum MK (2022). The 2021 WHO classification of tumors of the central nervous system: an update on pediatric low-grade gliomas and glioneuronal tumors. Brain Pathol Zurich Switz.

[6] Bale TA, Sait SF, Benhamida J, Ptashkin R, Haque S, Villafania L, Sill M, Sadowska J, Akhtar RB, Liechty B, Juthani R, Ladanyi M, Fowkes M, Karajannis MA, Rosenblum MK (2021). Malignant transformation of a polymorphous low grade neuroepithelial tumor of the young (PLNTY). Acta Neuropathol (Berl).

[7] Ballester LY, Moghadamtousi SZ, Leeds NE, Huse JT, Fuller GN (2019). Coexisting FGFR3 p.K650T mutation in two FGFR3-TACC3 fusion glioma cases. Acta Neuropathol Commun.

[8] Bielle F, Di Stefano A-L, Meyronet D, Picca A, Villa C, Bernier M, Schmitt Y, Giry M, Rousseau A, Figarella-Branger D, Maurage C-A, Uro-Coste E, Lasorella A, Iavarone A, Sanson M, Mokhtari K (2018). Diffuse gliomas with FGFR3-TACC3 fusion have characteristic histopathological and molecular features. Brain Pathol.

[9] Broggi G, Certo F, Altieri R, Caltabiano R, Gessi M, Barbagallo GMV (2021). A “polymorphous low-grade neuroepithelial tumor of the young (PLNTY)” diagnosed in an adult. Report of a case and review of the literature. Surg Neurol Int.

[10] Capper D, Jones DTW, Sill M, Hovestadt V, Schrimpf D, Sturm D, Koelsche C, Sahm F, Chavez L, Reuss DE, Kratz A, Wefers AK, Huang K, Pajtler KW, Schweizer L, Stichel D, Olar A, Engel NW, Lindenberg K, Harter PN, Braczynski AK, Plate KH, Dohmen H, Garvalov BK, Coras R, Hölsken A, Hewer E, Bewerunge-Hudler M, Schick M, Fischer R, Beschorner R, Schittenhelm J, Staszewski O, Wani K, Varlet P, Pages M, Temming P, Lohmann D, Selt F, Witt H, Milde T, Witt O, Aronica E, Giangaspero F, Rushing E, Scheurlen W, Geisenberger C, Rodriguez FJ, Becker A, Preusser M, Haberler C, Bjerkvig R, Cryan J, Farrell M, Deckert M, Hench J, Frank S, Serrano J, Kannan K, Tsirigos A, Brück W, Hofer S, Brehmer S, Seiz-Rosenhagen M, Hänggi D, Hans V, Rozsnoki S, Hansford JR, Kohlhof P, Kristensen BW, Lechner M, Lopes B, Mawrin C, Ketter R, Kulozik A, Khatib Z, Heppner F, Koch A, Jouvet A, Keohane C, Mühleisen H, Mueller W, Pohl U, Prinz M, Benner A, Zapatka M, Gottardo NG, Driever PH, Kramm CM, Müller HL, Rutkowski S, von Hoff K, Frühwald MC, Gnekow A, Fleischhack G, Tippelt S, Calaminus G, Monoranu C-M, Perry A, Jones C, Jacques TS, Radlwimmer B, Gessi M, Pietsch T, Schramm J, Schackert G, Westphal M, Reifenberger G, Wesseling P, Weller M, Collins VP, Blümcke I, Bendszus M, Debus J, Huang A, Jabado N, Northcott PA, Paulus W, Gajjar A, Robinson GW, Taylor MD, Jaunmuktane Z, Ryzhova M, Platten M, Unterberg A, Wick W, Karajannis MA, Mittelbronn M, Acker T, Hartmann C, Aldape K, Schüller U, Buslei R, Lichter P, Kool M, Herold-Mende C, Ellison DW, Hasselblatt M, Snuderl M, Brandner S, Korshunov A, von Deimling A, Pfister SM (2018). DNA methylation-based classification of central nervous system tumours. Nature.

[11] Capper D, Stichel D, Sahm F, Jones DTW, Schrimpf D, Sill M, Schmid S, Hovestadt V, Reuss DE, Koelsche C, Reinhardt A, Wefers AK, Huang K, Sievers P, Ebrahimi A, Schöler A, Teichmann D, Koch A, Hänggi D, Unterberg A, Platten M, Wick W, Witt O, Milde T, Korshunov A, Pfister SM, von Deimling A (2018). Practical implementation of DNA methylation and copy-number-based CNS tumor diagnostics: the Heidelberg experience. Acta Neuropathol (Berl).

[12] Chen Y, Tian T, Guo X, Zhang F, Fan M, Jin H, Liu D (2020). Polymorphous low-grade neuroepithelial tumor of the young: case report and review focus on the radiological features and genetic alterations. BMC Neurol.

[13] Di Stefano AL, Picca A, Saragoussi E, Bielle F, Ducray F, Villa C, Eoli M, Paterra R, Bellu L, Mathon B, Capelle L, Bourg V, Gloaguen A, Philippe C, Frouin V, Schmitt Y, Lerond J, Leclerc J, Lasorella A, Iavarone A, Mokhtari K, Savatovsky J, Alentorn A, Sanson M, TARGET study group (2020). Clinical, molecular, and radiomic profile of gliomas with FGFR3-TACC3 fusions. Neuro-Oncol.

[14] Farouk Sait S, Gilheeney SW, Bale TA, Haque S, Dinkin MJ, Vitolano S, Rosenblum MK, Ibanez K, Prince DE, Spatz KH, Dunkel IJ, Karajannis MA (2021). Debio1347, an oral FGFR inhibitor: results from a single-center study in pediatric patients with recurrent or refractory FGFR-Altered gliomas. JCO Precis Oncol.

[15] Ferguson SD, Zhou S, Huse JT, de Groot JF, Xiu J, Subramaniam DS, Mehta S, Gatalica Z, Swensen J, Sanai N, Spetzler D, Heimberger AB (2018). Targetable gene fusions associate with the IDH wild-type astrocytic lineage in adult gliomas. J Neuropathol Exp Neurol.

[16] Granberg KJ, Annala M, Lehtinen B, Kesseli J, Haapasalo J, Ruusuvuori P, Yli-Harja O, Visakorpi T, Haapasalo H, Nykter M, Zhang W (2017). Strong FGFR3 staining is a marker for FGFR3 fusions in diffuse gliomas. Neuro-Oncology.

[17] Huse JT, Snuderl M, Jones DTW, Brathwaite CD, Altman N, Lavi E, Saffery R, Sexton-Oates A, Blumcke I, Capper D, Karajannis MA, Benayed R, Chavez L, Thomas C, Serrano J, Borsu L, Ladanyi M, Rosenblum MK (2017). Polymorphous low-grade neuroepithelial tumor of the young (PLNTY): an epileptogenic neoplasm with oligodendroglioma-like components, aberrant CD34 expression, and genetic alterations involving the MAP kinase pathway. Acta Neuropathol (Berl).

[18] Johnson A, Severson E, Gay L, Vergilio J-A, Elvin J, Suh J, Daniel S, Covert M, Frampton GM, Hsu S, Lesser GJ, Stogner-Underwood K, Mott RT, Rush SZ, Stanke JJ, Dahiya S, Sun J, Reddy P, Chalmers ZR, Erlich R, Chudnovsky Y, Fabrizio D, Schrock AB, Ali S, Miller V, Stephens PJ, Ross J, Crawford JR, Ramkissoon SH (2017). Comprehensive genomic profiling of 282 pediatric low- and high-grade gliomas reveals genomic drivers, tumor mutational burden, and hypermutation signatures. Oncologist.

[19] Lassman AB, Sepúlveda-Sánchez JM, Cloughesy TF, Gil-Gil MJ, Puduvalli VK, Raizer JJ, De Vos FYF, Wen PY, Butowski NA, Clement PMJ, Groves MD, Belda-Iniesta C, Giglio P, Soifer HS, Rowsey S, Xu C, Avogadri F, Wei G, Moran S, Roth P (2022). Infigratinib in patients with recurrent gliomas and FGFR alterations: a multicenter phase II study. Clin Cancer Res Off J Am Assoc Cancer Res.

[20] Linzey JR, Marini B, McFadden K, Lorenzana A, Mody R, Robertson PL, Koschmann C (2017). Identification and targeting of an FGFR fusion in a pediatric thalamic “central oligodendroglioma. NPJ Precis Oncol.

[21] Louis DN, Perry A, Wesseling P, Brat DJ, Cree IA, Figarella-Branger D, Hawkins C, Ng HK, Pfister SM, Reifenberger G, Soffietti R, von Deimling A, Ellison DW (2021). The 2021 WHO classification of tumors of the central nervous system: a summary. Neuro-Oncology.

[22] Mata DA, Benhamida JK, Lin AL, Vanderbilt CM, Yang S-R, Villafania LB, Ferguson DC, Jonsson P, Miller AM, Tabar V, Brennan CW, Moss NS, Sill M, Benayed R, Mellinghoff IK, Rosenblum MK, Arcila ME, Ladanyi M, Bale TA (2020). Genetic and epigenetic landscape of IDH-wildtype glioblastomas with FGFR3-TACC3 fusions. Acta Neuropathol Commun.

[23] Métais A, Appay R, Pagès M, Gallardo C, Silva K, Siegfried A, Perbet R, Maurage C-A, Scavarda D, Fina F, Uro-Coste E, Riffaud L, Colin C, Figarella-Branger D, contributors of the Biopathology RENOCLIP-LOC network (2022). Low-grade epilepsy-associated neuroepithelial tumours with a prominent oligodendroglioma-like component: the diagnostic challenges. Neuropathol Appl Neurobiol.

[24] Pagès M, Debily M-A, Fina F, Jones DTW, Saffroy R, Castel D, Blauwblomme T, Métais A, Bourgeois M, Lechapt-Zalcman E, Tauziède-Espariat A, Andreiuolo F, Chrétien F, Grill J, Boddaert N, Figarella-Branger D, Beroukhim R, Varlet P (2022). The genomic landscape of dysembryoplastic neuroepithelial tumours and a comprehensive analysis of recurrent cases. Neuropathol Appl Neurobiol.

[25] Qaddoumi I, Orisme W, Wen J, Santiago T, Gupta K, Dalton JD, Tang B, Haupfear K, Punchihewa C, Easton J, Mulder H, Boggs K, Shao Y, Rusch M, Becksfort J, Gupta P, Wang S, Lee RP, Brat D, Peter Collins V, Dahiya S, George D, Konomos W, Kurian KM, McFadden K, Serafini LN, Nickols H, Perry A, Shurtleff S, Gajjar A, Boop FA, Klimo PD, Mardis ER, Wilson RK, Baker SJ, Zhang J, Wu G, Downing JR, Tatevossian RG, Ellison DW (2016). Genetic alterations in uncommon low-grade neuroepithelial tumors: BRAF, FGFR1, and MYB mutations occur at high frequency and align with morphology. Acta Neuropathol (Berl).

[26] Riva G, Cima L, Villanova M, Ghimenton C, Sina S, Riccioni L, Munari G, Fassan M, Giangaspero F, Eccher A (2018). Low-grade neuroepithelial tumor: unusual presentation in an adult without history of seizures. Neuropathology.

[27] Roux A, Tran S, Edjlali M, Saffroy R, Tauziede-Espariat A, Zanello M, Gareton A, Dezamis E, Dhermain F, Chretien F, Lechapt-Zalcman E, Oppenheim C, Pallud J, Varlet P (2021). Prognostic relevance of adding MRI data to WHO 2016 and cIMPACT-NOW updates for diffuse astrocytic tumors in adults. Working toward the extended use of MRI data in integrated glioma diagnosis. Brain Pathol Zurich Switz.

[28] Schittenhelm J, Ziegler L, Sperveslage J, Mittelbronn M, Capper D, Burghardt I, Poso A, Biskup S, Skardelly M, Tabatabai G (2020). FGFR3 overexpression is a useful detection tool for FGFR3 fusions and sequence variations in glioma. Neuro-Oncol Pract.

[29] Shibata N, Cho N, Koyama H, Naito M (2022). Development of a degrader against oncogenic fusion protein FGFR3-TACC3. Bioorg Med Chem Lett.

[30] Singh D, Chan JM, Zoppoli P, Niola F, Sullivan R, Castano A, Liu EM, Reichel J, Porrati P, Pellegatta S, Qiu K, Gao Z, Ceccarelli M, Riccardi R, Brat DJ, Guha A, Aldape K, Golfinos JG, Zagzag D, Mikkelsen T, Finocchiaro G, Lasorella A, Rabadan R, Iavarone A (2012). Transforming fusions of FGFR and TACC genes in human glioblastoma. Science.

[31] Stefano ALD, Fucci A, Frattini V, Labussiere M, Mokhtari K, Zoppoli P, Marie Y, Bruno A, Boisselier B, Giry M, Savatovsky J, Touat M, Belaid H, Kamoun A, Idbaih A, Houillier C, Luo FR, Soria J-C, Tabernero J, Eoli M, Paterra R, Yip S, Petrecca K, Chan JA, Finocchiaro G, Lasorella A, Sanson M, Iavarone A (2015). Detection, characterization, and inhibition of FGFR–TACC fusions in IDH wild-type glioma. Clin Cancer Res.

[32] Stichel D, Schrimpf D, Sievers P, Reinhardt A, Suwala AK, Sill M, Reuss DE, Korshunov A, Casalini BM, Sommerkamp AC, Ecker J, Selt F, Sturm D, Gnekow A, Koch A, Simon M, Driever PH, Schüller U, Capper D, van Tilburg CM, Witt O, Milde T, Pfister SM, Jones DTW, von Deimling A, Sahm F, Wefers AK (2021). Accurate calling of KIAA1549-BRAF fusions from DNA of human brain tumours using methylation array-based copy number and gene panel sequencing data. Neuropathol Appl Neurobiol.

[33] Stupp R, Hegi ME, Mason WP, van den Bent MJ, Taphoorn MJB, Janzer RC, Ludwin SK, Allgeier A, Fisher B, Belanger K, Hau P, Brandes AA, Gijtenbeek J, Marosi C, Vecht CJ, Mokhtari K, Wesseling P, Villa S, Eisenhauer E, Gorlia T, Weller M, Lacombe D, Cairncross JG, Mirimanoff R-O, European Organisation for Research and Treatment of Cancer Brain Tumour and Radiation Oncology Groups, National Cancer Institute of Canada Clinical Trials Group (2009). Effects of radiotherapy with concomitant and adjuvant temozolomide versus radiotherapy alone on survival in glioblastoma in a randomised phase III study: 5-year analysis of the EORTC-NCIC trial. Lancet Oncol.

[34] Wang Y, Liang D, Chen J, Chen H, Fan R, Gao Y, Gao Y, Tao R, Zhang H (2021). Targeted therapy with anlotinib for a patient with an oncogenic FGFR3-TACC3 fusion and recurrent glioblastoma. Oncologist.

[35] Wu Z, Lopes Abath Neto O, Bale TA, Benhamida J, Mata D, Turakulov R, Abdullaev Z, Marker D, Ketchum C, Chung H-J, Giannini C, Quezado M, Pratt D, Aldape K (2022). DNA methylation analysis of glioblastomas harboring FGFR3-TACC3 fusions identifies a methylation subclass with better patient survival. Acta Neuropathol (Berl).

[36] Zhang Y, Lucas C-HG, Young JS, Morshed RA, McCoy L, Oberheim Bush NA, Taylor JW, Daras M, Butowski NA, Villanueva-Meyer JE, Cha S, Wrensch M, Wiencke JK, Lee JC, Pekmezci M, Phillips JJ, Perry A, Bollen AW, Aghi MK, Theodosopoulos P, Chang EF, Hervey-Jumper SL, Berger MS, Clarke JL, Chang SM, Molinaro AM, Solomon DA (2022). Prospective genomically-guided identification of “early/evolving” and “undersampled” IDH-wildtype glioblastoma leads to improved clinical outcomes. Neuro-Oncology.

