# Supplementary material for: Clinico-pathological and epigenetic heterogeneity of diffuse gliomas with FGFR3::TACC3 fusion
Source: Acta Neuropathol Commun. 2023 Jan 16;11:14. doi: 10.1186/s40478-023-01506-z (PMC9843943; doi:10.1186/s40478-023-01506-z)
Supplement: Supplementary file 1 — Additional file 1: Table S1. Correlation table (Fisher’s exact tests). [file 40478_2023_1506_MOESM1_ESM.docx]

Additional file 1: Table S1

|  | Tumor location | Initial histological diagnosis | TERT promotor mutation status | t-SNE cluster | FGFR3(Ex17):  TACC3(Ex11) | FGFR3(Ex17):  TACC3(Ex10) |
| --- | --- | --- | --- | --- | --- | --- |
| Age at diagnosis | NS | **0.014** | **0.001** | **0.002** | **0.005** | **0.027** |
| Tumor Location | - | NS | NS | **0.017** | NS | NS |
| Initial histological diagnosis |  | - | 0.018 | 0.005 | NS | 0.074 |
| TERT promotor mutation status |  |  | - | **0.000** | **0.022** | **0.026** |
| t-SNE cluster |  |  |  | - | NS | NS |
| FGFR3(Ex17):  TACC3(Ex11) |  |  |  |  | - | **0.017** |
| FGFR3(Ex17):  TACC3(Ex10) |  |  |  |  |  | - |
